# Pure red cell aplasia associated with autoimmune hepatitis successfully treated with cyclosporine A

**DOI:** 10.1007/s12328-013-0448-0

**Published:** 2014-01-08

**Authors:** Akira Sato, Fumiaki Sano, Toshiya Ishii, Kayo Adachi, Ryujirou Negishi, Nobuyuki Matsumoto, Chiaki Okuse

**Affiliations:** 1 Division of Gastroenterology, Department of Internal Medicine, St. Marianna University School of Medicine Yokohama City Seibu Hospital, 1197-1 Yasashicho Asahi-ku, Yokohama, 241-0811 Japan; 2 Division of Hematology and Oncology, Department of Internal Medicine, St. Marianna University School of Medicine Yokohama City Seibu Hospital, 1197-1 Yasashicho Asahi-ku, Yokohama, 241-0811 Japan; 3 Division of Gastroenterology and Hepatology, Department of Internal Medicine, St. Marianna University School of Medicine, 2-16-1 Sugao, Miyamae-ku, Kawasaki, 216-8511 Japan

**Keywords:** Autoimmune hepatitis, Pure red cell aplasia, Cyclosporine

## Abstract

A 47-year-old female with a 17-year history of autoimmune hepatitis had been treated with prednisolone, azathioprine, and ursodeoxycholic acid. Although her alanine aminotransferase level occasionally showed mild abnormality, the prednisolone dose could not be increased because she had developed cataract during the course of her illness. In May 2012, she developed severe normochromic normocytic anemia without hemorrhage, and azathioprine was discontinued because it was suspected of being the cause. However, anemia recurred frequently even after discontinuation, necessitating repeated blood transfusions. Bone marrow analysis revealed selective erythroblastopenia, thus leading to a diagnosis of pure red cell aplasia. Cyclosporine A was administered, which led to a dramatic recovery from anemia, and stabilized her alanine aminotransferase levels. Furthermore, the prednisolone dose could be gradually tapered. Pure red cell aplasia associated with autoimmune hepatitis is extremely rare. The present case shows that patients with autoimmune hepatitis refractory to the standard treatment regimen and those with concomitant pure red cell aplasia may be treated with cyclosporine A.

## Introduction

Pure red cell aplasia (PRCA) is an acquired anemia that may be primary or secondary to a variety of neoplastic, autoimmune, or infectious diseases or to exposure to various drugs; most cases of PRCA are considered to be autoimmune-mediated [1]. Autoimmune hepatitis is a chronic liver inflammation that generally affects young-to-middle-aged women, and immune suppressants, particularly corticosteroids, are effective for treatment. Patients with autoimmune hepatitis occasionally have or acquire concomitant autoimmune diseases, but association with PRCA has rarely been reported. We report a case of PRCA complicated with autoimmune hepatitis that was treated with cyclosporine A (CsA) and exhibited favorable outcomes in managing both diseases.

## Case report

**Fig. 1:**
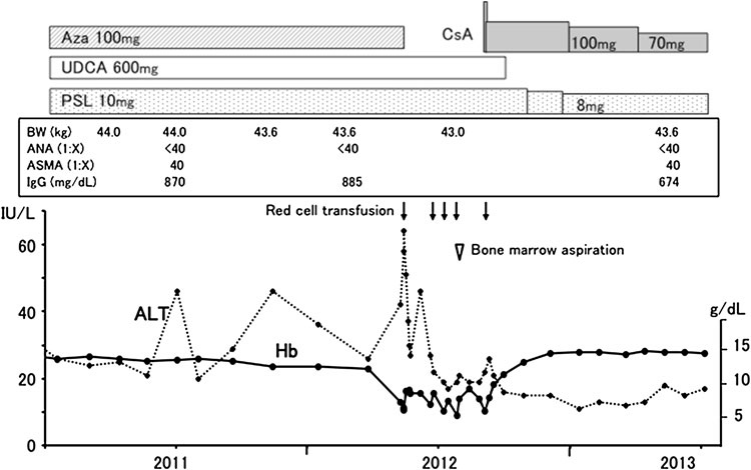
Clinical course of the patient in the present study. *Aza* azathioprine, *PSL* prednisolone, *UDCA* ursodeoxycholic acid, *CsA* cyclosporine A, *BW* body weight, *ANA* antinuclear antibody, *ASMA* anti-smooth muscle antibody, *IgG* immunoglobulin G, *ALT* alanine aminotransferase, *Hb* hemoglobin dosage of all medicines is expressed as dose/day

A 30-year-old female with acute hepatitis was referred to our hospital in 1995. She had been asymptomatic until the disease onset. She had no history of significant medical condition, blood transfusion, or daily intake of medicine and did not consume alcohol. Her serum alanine aminotransferase (ALT) level had fluctuated from 300 to 1116 IU/L for 3 months before admission. On admission, laboratory examinations revealed total bilirubin 2.5 mg/dL, aspartate transaminase (AST) 842 IU/L, ALT 956 IU/L, alkaline phosphatase 153 IU/L, γ-glutamyl transpeptidase 68 IU/L, γ-globulin 2.3 g/dL, and immunoglobulin G 2,710 mg/dL. Hepatitis B virus surface antigen and hepatitis C virus antibody were negative, antinuclear antibody was weakly positive (1:40, homogeneous), anti-smooth muscle antibody was positive (1:320), and anti-mitochondrial antibody was negative. Human leukocyte antigen was positive for DR-4. Histological analysis of the liver revealed marked interface hepatitis with rosette formation in the parenchyma. On the basis of these findings, she was diagnosed with autoimmune hepatitis and started on prednisolone (30 mg/day). Her serum ALT level gradually declined. Her prednisolone dose was tapered to 10–15 mg/day, which was administered with ursodeoxycholic acid (UDCA; 600 mg/day) to maintain her ALT level <40 IU/L. Since 2002, she had been treated by a doctor at home, and the aforementioned regimen was continued. In 2008, she developed steroid-induced cataract and was referred to our hospital to consult about treatment options for autoimmune hepatitis. At the time of referral, she was 150 cm in height and weighed 43.5 kg (body mass index 19.3 kg/m^2^), and she was being administered 12.5 mg/day of prednisolone and 600 mg/day of UDCA, and her serum AST and ALT levels were 23 and 34 IU/L, respectively. We added azathioprine (100 mg/day) to this regimen and gradually tapered the prednisolone dose to 10 mg/day; her ALT level was maintained between 22 and 40 IU/L with occasional mild flare-ups. In May 2012, she complained shortness of breath and easy fatigability and significant pallor was noted on physical examination. A hemogram revealed hemoglobin 6.4 g/dL, mean red cell volume 117.3 fL, reticulocyte count 0.6 %, white blood cell count 2,600 cells/μL, and platelet count 207,000 cells/μL. Laboratory serum analyses revealed total protein 6.0 g/dL, albumin 3.7 g/dL, AST 32 IU/L, ALT 42 IU/L, lactic acid dehydrogenase 221 IU/L, and normal bilirubin and alkaline phosphatase levels. Her serum iron level was 214 μg/dL, erythropoietin level was 3,750 mIU (normal 9.1–32.8 mIU), and vitamin B12 and folic acid levels were 307 pg/mL (normal 233–914 pg/mL) and 3.3 ng/mL (normal 3.6–12.9 ng/mL), respectively. She showed no symptoms of recent infection, such as fever or myalgia, and gastrointestinal endoscopy showed no evidence of hemorrhagic lesions. She was initially suspected of having azathioprine-induced anemia; therefore, azathioprine was discontinued. A red cell transfusion was performed, which increased her hemoglobin level to 8.3 g/dL; however, after 4 weeks, her hemoglobin levels decreased to 6.1 g/dL (Fig. 1). Another transfusion was performed; however, severe anemia kept recurring and repeat transfusions were required. Her reticulocyte count was consistently low (0.2–0.6 %) except just after transfusion. In July 2012, bone marrow aspiration was performed, which revealed hypocellular bone marrow with an absence of erythroblasts. Giant proerythroblast was not observed and her serum was negative for immunoglobulin (Ig)M antibody to parvovirus B19, although viral DNA was not examined. Therefore, she was diagnosed with acquired PRCA on the basis of these findings, and CsA was administered on September 2012 at an initial dose of 6 mg/kg/day (250 mg/day). After 7 weeks, her hemoglobin level and reticulocyte count both showed considerable improvement (12.4 g/dL and 2.4 %, respectively). The CsA dose was gradually reduced and maintained at 1.6 mg/kg/day (70 mg/day) since April 2013, without relapse of anemia. Her cyclosporine trough blood level has ranged from 69 to 88 ng/mL. In October 2012, UDCA was discontinued and the prednisolone dose was tapered to 9 mg/day; prednisolone has been maintained at 8 mg/day since January 2013. The mild fluctuations in serum AST and ALT levels noted after azathioprine discontinuation ceased after initiating CsA therapy. Her AST and ALT levels have remained <20 IU/L for 8 months since the initiation of therapy.

## Discussion

Our patient presented with acute-onset severe anemia with selective erythroblastopenia in the bone marrow. Therefore, she was diagnosed with PRCA. PRCA is a syndrome characterized by severe normochromic normocytic anemia, reticulocytopenia, and a striking erythroblastopenia in the bone marrow. PRCA is classified into congenital and acquired, and the latter is further classified into idiopathic and secondary to various infections, hematological malignancies, collagen vascular diseases, thymoma, and exposure to a variety of drugs and chemicals [1]. Acquired PRCA, except when caused by parvovirus B19 infection, is rare; its incidence in Japan is estimated to be 0.3/1.0 million persons/year [2]. Azathioprine is known to cause PRCA; because our patient had been receiving azathioprine for 4 years, we initially suspected it might be the cause of anemia. However, drug-induced PRCA remits soon after drug discontinuation [1], and azathioprine-induced PRCA has been reported only in renal transplant patients [3, 4] excluding one case [5]. Therefore, we concluded that azathioprine was an unlikely causative agent. In addition, PRCA develops in patients with various autoimmune diseases, and two cases of PRCA associated with autoimmune hepatitis were reported in 1978 and 1986. Fox et al. [6] reported a 37-year-old female patient who was treated with cyclophosphamide and splenectomy for anemia, but these interventions showed limited effectiveness for the disease. Therefore, the patient died 2 years after onset of PRCA. In contrast, the 54-year-old female patient reported by Trinchet et al. [7] recovered from PRCA with 2 months of cyclophosphamide therapy. The clinical findings of these 2 cases, including response to corticosteroid for hepatitis, were consistent with autoimmune hepatitis. However, human parvovirus B19 and hepatitis C virus infections may cause PRCA [1, 8], and these associations were not ruled out. Recently, it was reported that 1 % of cases with PRCA were associated with autoimmune hepatitis [9], but there are no published reports in which these virus associations were excluded.

The mechanism of selective erythroid hypoplasia in PRCA is poorly understood; however, most cases of chronic PRCA are considered to be mediated by diverse autoimmune mechanisms, such as antibodies or T cell- and NK cell-mediated, as reviewed by Fisch et al. [10]. Therefore, several immunosuppressive therapies have been used. Of them, CsA, a calcineurin inhibitor, suppresses the immune response by inhibiting the signal transduction pathway [11] and exhibits a favorable effect for PRCA [9]; it is now recommended as a first-line therapy for the disease [12]. On the other hand, the standard treatment for autoimmune hepatitis is corticosteroid with or without azathioprine [13–15]. Furthermore, the efficacy of UDCA has been reported in studies from Japan [16, 17], although negative results have also been reported [18]. Our patient had been treated with the three aforementioned drugs, but her transaminase levels were not fully controlled. For patients with autoimmune hepatitis refractory to standard therapy, the American Association for the Study of Liver Diseases and the European Association for the Study of Liver evaluated the efficacy of mycophenolate mofetil rather than CsA [13, 14]. However, the British Society of Gastroenterology (BSG) comments that CsA could be used as an alternative therapy in patients who fail to achieve complete biochemical or histological remission on standard therapy considering the balance of its toxicity profile and potential benefits [15]. CsA for autoimmune hepatitis was first used by Mistilis et al. [19], and subsequent trials confirmed its efficacy in managing autoimmune hepatitis. In one of them, 19 patients (10 with side-effects with or unresponsiveness to corticosteroid) were treated with 2–5 mg/kg/day (target trough levels 100–300 ng/mL) of CsA [20]. Of them, 15 patients completed 6 months of treatment with significant reductions in serum ALT and histological activity index scores were observed over the 6-month period; moreover, 14 patients achieved complete clinical remission. In another study, 32 children were treated with CsA as a first-line therapy with a starting dose of 4 mg/kg/day and adjusted to target trough levels 200–300 ng/mL in the first 3 months followed by 150–250 ng/mL; within 6 months of treatment, 78 % patients had normal ALT levels [21]. In both studies, clinical findings and laboratory tests including renal functions were monitored weekly or biweekly in the first 2 months, followed by monthly monitoring; in addition, there was no significant effect on serum creatinine level, and adverse effect related to CsA appeared mild and transient. Although no trial has evaluated long-term safety of CsA therapy in autoimmune hepatitis, Sciveres et al. [22] reported CsA treatment for 8–89 (median 35.6) months in 12 patients with autoimmune liver diseases, including 8 patients with autoimmune hepatitis (6 patients with side-effects or unresponsiveness to corticosteroid). The maintenance trough levels of CsA targeted to 100–150 ng/mL (50–100 ng/mL after 1 year), and complete remission was achieved in all patients without treatment withdrawal due to side-effects. Furthermore, in one Japanese patient, CsA was administered at 100 mg/day (1.67 mg/kg/day) for >6 years without side-effects leading to marked improvement of liver fibrosis [23]. Nephropathy is the most common side-effect of CsA; however, multivariate analysis in patients with various autoimmune diseases revealed that administering <5 mg/kg/day of CsA and avoidance of increase in serum creatinine of >30 % above the patient’s baseline value could minimize the development of nephropathy [24]. Thus, patients with autoimmune hepatitis who are intolerant to corticosteroid or azathioprine, or unresponsive to standard regimen, could be prescribed CsA with careful follow-up.

In our patient, CsA was administered by chance with the occurrence of PRCA and showed better efficacy than the former therapy of azathioprine plus UDCA, thereby enabling the prednisolone dose to be tapered. The prognosis for patients with PRCA is favorable showing >10 years of median survival time [25, 26]; nevertheless, the majority of the patients need maintenance therapy after remission because of frequent relapse due to stopping therapy. Considering that the mean maintenance dose of CsA to prevent relapse is reportedly 2.2 mg/kg for idiopathic PRCA [9] and 2.5 mg/kg for secondary PRCA [26], our patient might require continuation of CsA and lower doses of corticosteroid than that administered before PRCA onset to treat autoimmune hepatitis.

In conclusion, we presented a case of PRCA associated with autoimmune hepatitis, and CsA showed a favorable effect for both diseases. Therefore, CsA should be considered for managing patients with autoimmune hepatitis who are refractory or intolerant to the standard regimen. However, these patients should be monitored for renal toxicity; they may need adequate prevention for infection [27] and follow-up for the occurrence of malignancies during long-term CsA therapy.
